# Genotype-guided new approach for dose optimisation of hydroxychloroquine administration in Chinese patients with SLE

**DOI:** 10.1136/lupus-2023-000997

**Published:** 2023-11-22

**Authors:** Han Xie, Xin Wen, Yuchun Wang, Xuan Huang, Qing Shu, Dandan Wang, Linyu Geng, Ziyi Jin, Wei Shen, Weihong Ge, Yizhun Zhu, Lingyun Sun

**Affiliations:** 1 School of Pharmacy, Faculty of Medicine, Macau University of Science and Technology, Macau SAR, China; 2 Department of Rheumatology and Immunology, Nanjing Drum Tower Hospital, Nanjing, Jiangsu Province, China; 3 Department of Pharmacy, Nanjing Drum Tower Hospital, Nanjing, Jiangsu Province, China; 4 School of Basic Medicine and Clinical Pharmacy, China Pharmaceutical University, Nanjing, Jiangsu Province, China; 5 State Key Laboratory of Quality Research in Chinese Medicine & School of Pharmacy, Macau University of Science and Technology, Macau SAR, China

**Keywords:** Antirheumatic Agents, Autoimmune Diseases, Lupus Erythematosus, Systemic

## Abstract

**Objectives:**

The study aims to investigate the impact of gene polymorphisms on blood hydroxychloroquine (HCQ) concentrations in patients with SLE and provide guidelines for individualised care.

**Methods:**

489 Chinese patients with SLE taking HCQ for more than 3 months were collected in this study. The blood HCQ, desethylhydroxychloroquine (DHCQ) and desethylchloroquine concentrations were measured. The optimal blood concentration of HCQ was determined by receiver operating characteristic curve analysis. Single nucleotide polymorphisms of metabolic enzymes involved in HCQ metabolism were genotyped and the associations with treatment effects were investigated.

**Results:**

The cut-off value of HCQ was 559.67 ng/mL, with sensitivity and specificity values of 0.51 and 0.89, respectively. The TC and CC genotypes of CYP2C8 (rs7910936) were significantly related to the increase in blood HCQ concentrations, and the CYP2C8 (rs10882521) TT genotype was associated with lower blood HCQ concentrations. The DHCQ:HCQ ratio was highest in patients with the GG genotype of the CYP2D6*10 (rs1065852) polymorphism and lowest in those with the AA genotype. Patients with the CYP2C8 (rs7910936) CC genotype were more likely to achieve the optimal blood concentration (p=0.030) in HCQ 200 mg/day group and patients with the CYP2D6*10 (rs1065852) GG genotype were more likely to reach the optimal blood concentration (p=0.049) in 400 mg/day group.

**Conclusions:**

Our results suggest that the optimal blood concentration of HCQ measured approximately 12–18 hours after the last dosage may be between 500 and 600 ng/mL in Chinese patients with SLE. The observed variations in HCQ concentrations between individuals can potentially be attributed to genetic polymorphisms in CYP2D6*10 (rs1065852) and CYP2C8 (rs7910936 and rs10882521). Genotypical testing of patients and regular monitoring of blood levels are recommended for optimising HCQ dosage management in Chinese patients with SLE.

**Trial registration number:**

ChiCTR2300070628.

**WHAT IS ALREADY KNOWN ON THIS TOPIC**

- Hydroxychloroquine (HCQ) blood concentrations vary widely between patients, even when taking the same dose at the same frequency.
- The blood HCQ concentration is closely related to the treatment response of SLE.

**WHAT THIS STUDY ADDS**

- Our study supports HCQ blood levels at 500–600 ng/mL to be clinically meaningful and statistically significant to predict efficacy (SLE Disease Activity Index ≤4) for Chinese patients with SLE.
- Polymorphisms in CYP2D6*10 (rs1065852) and CYP2C8 (rs7910936 and rs10882521) might explain the significant differences in whole-blood HCQ concentrations between individuals.

**HOW THIS STUDY MIGHT AFFECT RESEARCH, PRACTICE OR POLICY**

- The optimal concentration provides reference for clinical practice.
- Genotypical testing of patients and regular monitoring of blood levels may help to optimise HCQ dosing in Chinese patients with SLE.

## Introduction

Hydroxychloroquine (HCQ), a primary long-term therapy drug for SLE,^1 2^ can reduce disease activity, decrease damage accumulation and improve patient survival.^3 4^ The concentration of HCQ was closely related to clinical efficacy.^5^ Maintaining adequate HCQ blood concentration had an essential impact on preventing SLE flares, reducing complications risk and improving relapse rates.^6 7^ The available studies on the target therapeutic level of HCQ in patients with SLE have provided some guidance on clinical dosing, but they were still controversial and lacked studies in the Chinese population with SLE.^8 9^

HCQ has significant pharmacokinetic variability, showing large differences in blood concentrations between patients even when taking the same dose. This interindividual difference has not been fully understood. Pharmacogenetic polymorphisms can affect the rate of HCQ metabolism in vivo and may be an important reason for the differences in blood levels.^10 11^ HCQ is metabolised by cytochrome P450 (CYP450) enzymes (CYP2D6, CYP2C8, CYP3A4 and CYP3A5) into three products, including desethylhydroxychloroquine (DHCQ), desethylchloroquine (DCQ) and bisdeethylchloroquine, of which DHCQ is the main active metabolite of HCQ.^12 13^ Specific single nucleotide polymorphisms (SNPs) in CYP450 enzymes may impact HCQ activity, leading to differences in drug concentrations in patients with SLE. So far, few studies have investigated associations of genetic polymorphisms with blood concentrations of HCQ and its metabolites in Chinese patients with SLE. The association of polymorphisms in CYP2D6, CYP3A4 and CYP3A5 with HCQ blood levels has been investigated in some studies. For example, a study revealed the DHCQ:HCQ ratio was associated with CYP2D6*10 (rs1065852 and rs1135840) in Korean patients with lupus.^14^ However, few studies about CYP2C8 polymorphism were reported, and little is known about the effect of CYP polymorphisms on the concentrations of HCQ and its metabolites in Chinese patients with SLE.^15^

In this study, we determined the optimal blood concentration and analysed the relationship between the polymorphisms of CYP450 enzymes and blood concentrations of HCQ and its metabolites in Chinese patients with SLE. In addition, we investigated the influence of SNPs on SLE Disease Activity Index (SLEDAI) and the optimal blood concentration. We hope to provide guidelines for HCQ for individual care to optimise HCQ dosing.

## Patients and methods

### Study design and population

This study is a single-centre, prospective, observational clinical research. The study population was hospitalised patients with SLE from Nanjing Drum Tower Hospital from January 2020 to December 2022. The inclusion criteria were as follows: (1) diagnosed with SLE according to the 1997 American College of Rheumatology Classification Criteria^16^; (2) ages from 18 to 70 years old; (3) need treatment with oral HCQ (dose of HCQ was no more than 5 mg/kg/day, the maximum daily dose was 400 mg) for more than 3 months; (4) complete clinical data and relevant examination results; (5) patients agreed to participate in the study. The exclusion criteria were as follows: (1) allergy to HCQ or any identified contraindications to HCQ; (2) combined with other autoimmune diseases, such as rheumatoid arthritis, systemic sclerosis and mixed connective tissue diseases; (3) patients with a history of head and neck radiotherapy, hepatitis C, AIDS activity, sarcoidosis, Graves’ disease and diabetes history; or combined use of anticholinergics and other factors that may affect the diagnosis of the disease; (4) patients who were excluded from the combination of amiodarone, quinidine, propafenone, propranolol, cimetidine, fluoxetine, clarithromycin, itraconazole, ketoconazole, indinavir and other hepatic enzyme-inhibitory drugs or drugs that might affect blood routine examination such as Antler and Diyushengbai tablets; (5) patients with severe organ injury or with an estimated survival period of ≤3 months; (6) patients with SLE at risk and need to use high-dose glucocorticoids (≥1 mg/kg/day prednisone or equivalent doses of other glucocorticoids); (7) patients with malignant tumours; (8) patients with concurrent infections (including those with current symptoms and previous tuberculosis); (9) patients with pregnancy and lactation; (10) patients with insufficient or contaminated blood samples. Exit standard: (1) subjects decided to withdraw informed consent for any reason; (2) although the subjects did not explicitly withdraw from the study, they were no longer subjected to medication and testing and lost follow-up; (3) any pathological events, clinical adverse events or changes in the physical condition of the subjects led to researchers believe that continuing to participate in the study was not in the best interests of patients; (4) pregnancy events during the study; (5) other causes leading to the withdrawal of the study; (6) if the participants withdraw from the study before the end of the study, the reasons for withdrawal need to be recorded. The specific flow chart is shown in online supplemental figure 1.

10.1136/lupus-2023-000997.supp1 Supplementary data

After a period of long-term HCQ administration (>3 months), patients’ morning fasting venous blood samples were collected when they came to the hospital to do a follow-up check. The blood samples were generally obtained about 12–18 hours after last dosage. The whole-blood HCQ, DHCQ and DCQ concentrations were determined and SNPs of metabolic enzymes involved in HCQ metabolism were genotyped. At the time point of the patients’ initial dosing and sampling, the SLEDAI scores of patients were evaluated and the following laboratory tests were collected: white blood cell (WBC), platelet (PLT), red blood cell, C reactive protein, C3, C4, IgG, IgA, IgM, erythrocyte sedimentation rate, anti-double-stranded DNA (anti-dsDNA) and ANA.

### Measurement of HCQ, DHCQ and DCQ concentrations

We used a Shimadzu high-performance liquid chromatography device (LC-20A, Japan) to detect the concentrations of HCQ and its two primary metabolites (DHCQ and DCQ) in the whole blood of patients with SLE at Nanjing Drum Tower Hospital Precision Medicine Center (Nanjing Drum Tower Hospital, Nanjing, China), which included an LC-20AD pump, a SIL-20AC autosampler and RF-20A fluorescence detector. A YMC-Triart C18 column (250×4.6 mm, 5 µm) was used with a column temperature of 40°C and a 1 mL/min flow rate. The detection wavelength of the fluorescence detector was 337 nm for excitation and 405 nm for emission. Chloroquine was used as the internal standard. The peak areas of HCQ, DHCQ, DCQ and the internal standard in the chromatogram were recorded, and the concentrations of HCQ, DHCQ and DCQ were calculated according to the standard internal method.^17^ For all analytes (HCQ, DHCQ and DCQ), accuracies and imprecision values were within the acceptance criteria of ±15% of the mean concentrations. The lower limit of quantification was 3 ng/mL for HCQ and its two metabolites. Patients with HCQ <100 ng/mL were excluded for non-compliant with medication.^18^

### Genotyping and quality control

According to previous research, we selected important SNPs associated with HCQ metabolism.^14 19 20^ The inclusion criteria were as follows: (a) having a minor allele frequency (MAF) ≥0.05 in the Chinese population; (b) consistent with Hardy‐Weinberg equilibrium (HWE) (p≥0.05); (c) with genotyping rate ≥90%. As a result, CYP2C8 (rs7910936, rs10882521, rs17110453), CYP2D6 (rs1065852, rs1135840), CYP3A4 (rs2242480, rs2246709, rs4646440) and CYP3A5 (rs4646450, rs4646453) for a total of 10 SNPs were selected. DNA was extracted from the blood samples using the Wizard Genomic DNA purification Kit (Promega), and the DNA was stored at −20°C for quality control. PCR amplification primers and single base extension primers for the SNPs to be tested were designed using Agena’s website and were synthesised. The genotyping was implemented on an Agena MassARRAY system based on matrix-assisted laser desorption/ionisation time-of-flight mass spectrometry. The results were typed and output using TYPER V.4.0 software (Agena).

### Statistical analysis

Data were analysed using IBM SPSS Statistics (V.26, IBM Corporation). Independent-samples t-test and a general linear model were used to compare quantitative data. The Χ^2^ was used to analyse the qualitative data. Receiver operating characteristic (ROC) curve analysis was applied to find the optimal blood concentration of HCQ, and the cut-off value was determined by calculating the highest Jorden index associated with the best sensitivity and specificity. Logistic regression analysis was performed to evaluate the relationship between SNPs and the optimal blood HCQ concentration. Differences were considered statistically significant at p<0.05.

## Results

### Patient characteristics

**Figure 1:**
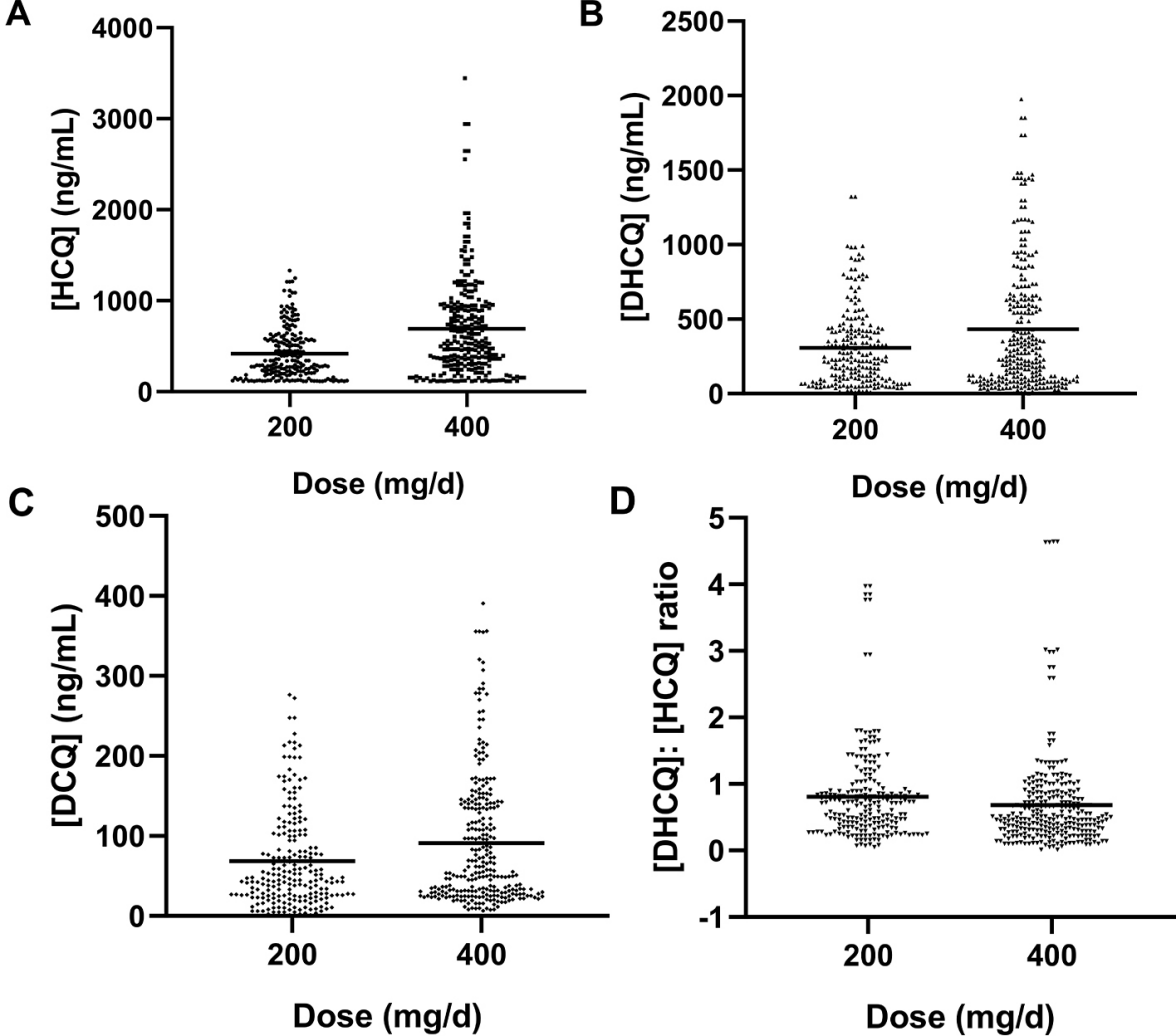
Correlation between the daily dose groups and concentrations of HCQ and its metabolites. (A) HCQ concentration; (B) DHCQ concentration; (C) DCQ concentration; (D) DHCQ-to-HCQ concentration ratio. DCQ, desethylchloroquine; DHCQ, desethylhydroxychloroquine; HCQ, hydroxychloroquine.

A total of 489 Chinese patients with SLE taking HCQ for more than 3 months were collected in this study. The recommended dose of HCQ was no more than 5 mg/kg/day. Patients were grouped by dose (200 mg/day or 400 mg/day). The demographic characteristics of the patients are as shown in table 1. For younger patients with shorter duration of treatment and a higher positive rate of ANA and anti-dsDNA, the dose of 400 mg/day was more common. In addition, patients taking HCQ 400 mg/day had higher blood levels and lower DHCQ-to-HCQ concentration ratio (table 1 and figure 1).

**Table 1:** Demographic characteristics of the patients

Category	Total population	200 mg/day	400 mg/day	P value
	(**n=489**)	(**n=217**)	(**n=272**)	**(200 vs 400 mg/day**)
Sex, no (%) female	422 (86.30)	182 (83.87)	240 (88.24)	0.163
Age, years, mean (SD)	44.18 (15.39)	48.62 (15.24)	40.63 (14.60)	**<0.001**
Body weight, kg, mean (SD)	59.49 (12.39)	58.79 (11.28)	60.07 (13.20)	0.256
BMI, kg/m^2^, mean (SD)	22.57 (3.97)	22.45 (3.57)	22.67 (4.26)	0.546
Duration of HCQ treatment, months, mean (SD)	12.18 (10.79)	14.77 (10.65)	10.12 (10.47)	**<0.001**
Receiving concomitant glucocorticoids, no (%)	371 (70.35)	159 (73.27)	212 (77.94)	0.231
Receiving concomitant immunosuppressants, no (%)	221 (40.49)	105 (48.39)	116 (42.65)	0.205
ANA positive, no (%)	433 (88.55)	178 (82.03)	255 (93.75)	**<0.001**
Anti-dsDNA positive, no (%)	216 (44.17)	85 (39.17)	131 (48.16)	**0.047**
SLEDAI before HCQ treatment, mean (SD)	8.01 (3.74)	8.13 (3.89)	7.92 (3.63)	0.539
HCQ, ng/mL, mean (SD)	569.38 (465.27)	417.06 (282.49)	690.90 (541.12)	**<0.001**
DHCQ, ng/mL, mean (SD)	376.56 (370.26)	307.28 (261.72)	431.84 (430.53)	**<0.001**
DCQ, ng/mL, mean (SD)	80.99 (73.37)	68.64 (60.63)	90.85 (80.90)	**0.001**
DHCQ:HCQ ratio, mean (SD)	0.74 (0.71)	0.81 (0.70)	0.68 (0.72)	**0.048**

Significant p values are in bold.

anti-dsDNA, anti-double-stranded DNA; BMI, body mass index; DCQ, desethylchloroquine; DHCQ, desethylhydroxychloroquine; HCQ, hydroxychloroquine; SLEDAI, SLE Disease Activity Index.

Online supplemental table 1 shows the comparison of clinical characteristics before and after long-term administration of HCQ. Most patients received HCQ combined with low-dose glucocorticoids and immunosuppressants during the treatment, with no significant changes (p=0.052 and p=0.137, respectively). However, after the administration of HCQ, the haematological indicators and inflammatory indicators such as C3, C4, IgG and IgA changed significantly. SLEDAI score decreased from 8.01±3.74 to 3.88±2.19.

### Determination of the optimal blood drug concentration

**Figure 2:**
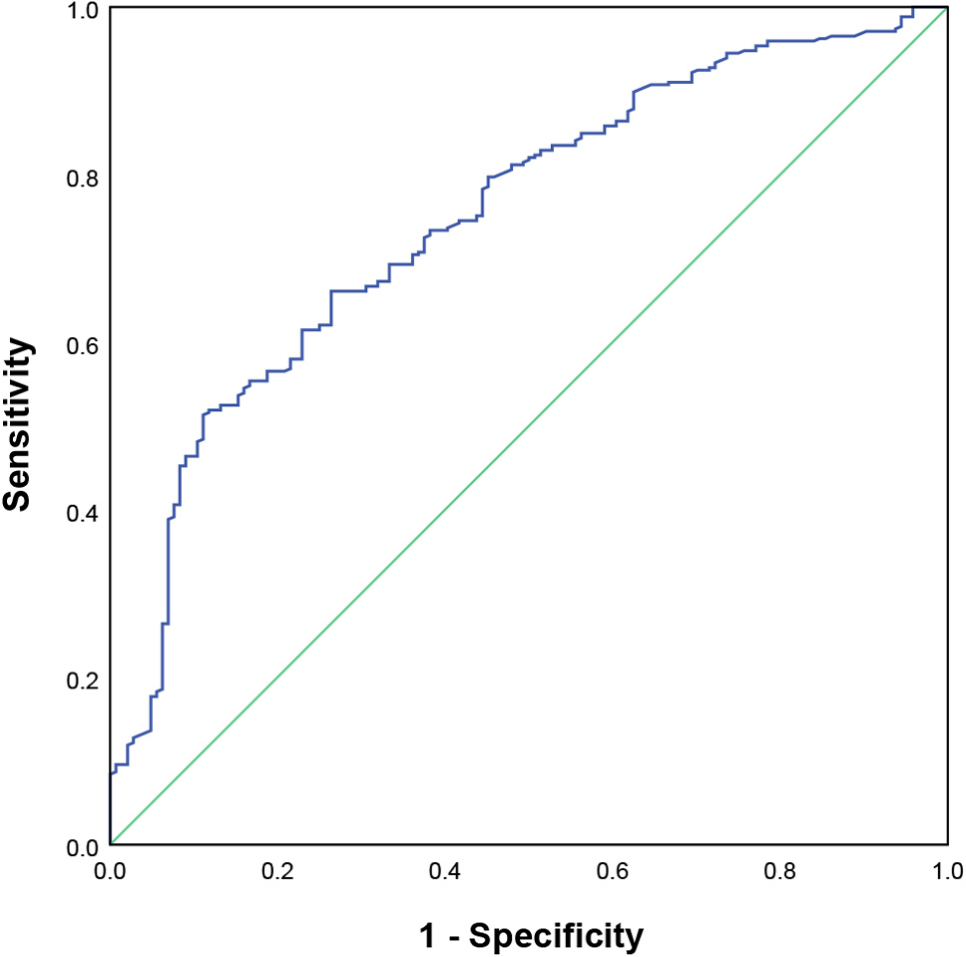
ROC curve of HCQ concentration in predicting efficacy. Positive was defined as SLEDAI ≤4. Cut-off value: 559.67 ng/mL; sensitivity: 0.51; specificity: 0.89; area under the curve: 0.75. HCQ, hydroxychloroquine; ROC, receiver operating characteristic; SLEDAI, SLE Disease Activity Index.

SLEDAI can effectively reflect the disease status of patients with SLE, and patients have low disease activity when SLEDAI ≤4.^21^ Therefore, the effective group of HCQ was defined by SLEDAI ≤4, and the ineffective group was SLEDAI >4. In our study, treatment was effective in 345 patients and ineffective in 144. Comparison of patients’ characteristics in effective and ineffective groups was listed in online supplemental table 2. ROC curve analysis was used to determine the optimal blood drug concentration of HCQ and its metabolites in predicting efficacy (online supplemental table 3, figure 2 and online supplemental figure 2). The area under the curve (AUC) of HCQ was 0.75, with sensitivity and specificity values of 0.51 and 0.89, respectively. The cut-off value of 559.67 ng/mL for HCQ was the optimum blood concentration obtained. The patients were then divided into two groups based on the optimum blood concentration: a high concentration group (≥559.67 ng/mL) and a low concentration group (<559.67 ng/mL), and clinical indicators were compared (online supplemental table 4). The results showed significant differences in PLT, C4, IgM, SLEDAI and the positive rate of anti-dsDNA between the two groups (p<0.05). Patients in the high concentration group exhibited elevated levels of PLT and C4, decreased levels of IgM and SLEDAI, and a lower positive rate of anti-dsDNA. Among patients who reached the optimal blood drug concentration, 91.74% of patients were treated effectively (SLEDAI ≤4).

10.1136/lupus-2023-000997.supp2 Supplementary data

10.1136/lupus-2023-000997.supp3 Supplementary data

10.1136/lupus-2023-000997.supp4 Supplementary data

To avoid the effects of comorbid medications, we performed stratified analyses (online supplemental table 5). Forty-nine patients were not combined with glucocorticoids or immunosuppressants during the treatment process. We conducted ROC analysis in these patients and obtained a cut-off value of 561.12 ng/mL (sensitivity=0.67, specificity=0.95, AUC=0.80) for the blood concentration of HCQ. Four hundred twenty-six patients used glucocorticoids in combination, and a cut-off value of 540.71 ng/mL (sensitivity=0.55, specificity=0.82, AUC=0.67) was obtained. Besides, the cut-off value was 502.10 ng/mL (sensitivity=0.58, specificity=0.90, AUC=0.79) for 256 patients who used immunosuppressants in combination. The main immunosuppressive agents used in this study included mycophenolate mofetil and tacrolimus. The cut-off value was 523.89 ng/mL for mycophenolate mofetil group and 535.10 ng/mL for tacrolimus group. Combined with the results of the stratified analysis, optimal blood levels of HCQ may be between 500 and 600 ng/mL.

### Relationship between gene polymorphisms and blood concentrations of HCQ and its metabolites

Genotypes and allele frequencies of 10 SNPs consistent with HWE (p>0.05) in 489 patients with SLE are shown in table 2. After adjusting by weight, age and duration of HCQ treatment, we found the polymorphisms of CYP2D6*10 (rs1065852) and CYP2C8 (rs7910936 and rs10882521) were significantly associated with HCQ and its metabolites in HCQ 200 and 400 mg/day group (table 3). In patients taking HCQ 200 mg/day, polymorphisms of CYP2C8 (rs7910936 and rs10882521) were associated with HCQ and DCQ. The C allele of CYP2C8 (rs7910936) was related to higher HCQ (p=0.001) and DCQ (p=0.014). The T allele of CYP2C8 (rs10882521) was related to lower HCQ (p=0.009) and DCQ (p=0.008). In patients with HCQ 400 mg/day, polymorphisms of CYP2C8 (rs7910936 and rs10882521) were associated with HCQ, DHCQ and DCQ. The C allele of CYP2C8 (rs7910936) was significantly associated with higher HCQ (p=0.004), DHCQ (p=0.004) and DCQ (p=0.005). The CYP2C8 (rs10882521) TT genotype had lower HCQ (p=0.003), DHCQ (p=0.009) and DCQ (p=0.001). In addition, polymorphism of CYP2D6*10 (rs1065852) was significantly correlated with the DHCQ:HCQ ratio in both dose groups (p=0.032 and p=0.036, respectively), and the DHCQ:HCQ ratio was lower in patients with the AA genotype compared with the GG and AG genotypes.

**Table 2:** Genotypes and allele frequencies of 10 SNPs in 489 Chinese patients with SLE

Polymorphism	Genotype	n (%)	Allele frequency	HWE p value
CYP2C8 (rs7910936)	TT	106 (21.86)	T=47.94%	0.321
	TC	253 (52.16)	C=52.06%	
	CC	126 (25.98)		
CYP2C8 (rs10882521)	GG	153 (31.42)	G=56.78%	0.462
	GT	247 (50.72)	T=43.22%	
	TT	87 (17.86)		
CYP2C8 (rs17110453)	AA	197 (40.96)	A=63.83%	0.835
	AC	220 (45.74)	C=36.17%	
	CC	64 (13.30)		
CYP3A4*1G (rs2242480)	CC	256 (53.00)	C=72.25%	0.385
	CT	186 (38.51)	T=27.75%	
	TT	41 (8.49)		
CYP3A4 (rs2246709)	GG	100 (20.53)	G=44.45%	0.491
	GA	233 (47.85)	A=55.55%	
	AA	154 (31.62)		
CYP3A4 (rs4646440)	GG	287 (59.67)	G=76.72%	0.317
	GA	164 (34.10)	A=23.28%	
	AA	30 (6.23)		
CYP3A5 (rs4646450)	GG	248 (51.03)	G=72.02%	0.361
	GA	204 (41.97)	A=27.98%	
	AA	34 (7.00)		
CYP3A5 (rs4646453)	CC	266 (54.62)	C=74.84%	0.101
	CA	197 (40.45)	A=25.16%	
	AA	24 (4.93)		
CYP2D6*10 (rs1065852)	AA	84 (17.29)	A=44.14%	0.054
	AG	261 (53.70)	G=55.86%	
	GG	142 (29.01)		
CYP2D6*10 (rs1135840)	GG	224 (46.38)	G=66.67%	0.056
	GC	196 (40.58)	C=33.33%	
	CC	63 (13.04)		

HWE, Hardy‐Weinberg equilibrium; SNPs, single nucleotide polymorphisms.

**Table 3:** Associations of SNP genotypes with blood concentrations of HCQ and its metabolites

	HCQ (95% CI)	DHCQ (95% CI)	DCQ (95% CI)	DHCQ:HCQ (95% CI)
200 mg/day				
CYP2C8 (rs7910936)				
ΤΤ	294.27 (214.36 to 374.18)	248.82 (172.56 to 325.08)	50.67 (33.13 to 68.21)	1.00 (0.79 to 1.21)
ΤC	430.38 (379.11 to 481.65)	309.95 (261.02 to 358.88)	68.23 (56.98 to 79.49)	0.79 (0.66 to 0.92)
ΤC	493.31 (424.45 to 562.17)	355.93 (290.21 to 421.65)	85.37 (70.26 to 100.49)	0.72 (0.54 to 0.90)
Ρ value	**0.001**	0.115	**0.014**	0.127
CYP2C8 (rs10882521)				
GG	473.90 (410.74 to 537.06)	360.81 (301.27 to 420.35)	85.90 (72.17 to 99.63)	0.79 (0.62 to 0.95)
GT	422.25 (369.07 to 475.43)	297.02 (246.89 to 347.16)	62.83 (51.27 to 74.39)	0.75 (0.61 to 0.89)
TT	309.57 (226.44 to 392.71)	241.95 (163.59 to 320.32)	53.54 (35.46 to 71.61)	0.99 (0.78 to 1.21)
P value	**0.009**	0.053	**0.008**	0.169
CYP2C8 (rs17110453)				
AA	444.37 (384.44 to 504.31)	330.66 (274.43 to 386.90)	74.34 (61.18 to 87.50)	0.76 (0.61 to 0.91)
AC	429.33 (374.91 to 483.75)	306.81 (255.75 to 357.86)	67.97 (56.02 to 79.92)	0.78 (0.65 to 0.92)
CC	329.88 (227.97 to 431.80)	262.00 (166.39 to 357.61)	60.02 (37.64 to 82.40)	1.00 (0.75 to 1.26)
P value	0.155	0.468	0.524	0.264
CYP3A4*1G (rs2242480)				
CC	391.67 (335.84 to 447.50)	285.96 (234.15 to 337.76)	69.46 (57.21 to 81.70)	0.89 (0.75 to 1.03)
CT	452.24 (396.51 to 507.96)	348.62 (296.92 to 400.32)	67.00 (54.78 to 79.23)	0.80 (0.66 to 0.94)
TT	395.38 (285.55 to 505.22)	252.92 (151.00 to 354.83)	73.93 (49.84 to 98.03)	0.59 (0.31 to 0.87)
P value	0.289	0.123	0.873	0.174
CYP3A4 (rs2246709)				
GG	447.88 (366.58 to 529.17)	366.08 (290.74 to 441.43)	69.38 (51.63 to 87.12)	0.73 (0.53 to 0.94)
GA	410.40 (359.49 to 461.31)	298.98 (251.80 to 346.17)	66.30 (55.19 to 77.41)	0.80 (0.67 to 0.93)
AA	412.87 (337.95 to 487.79)	286.15 (216.71 to 355.58)	74.06 (57.70 to 90.41)	0.92 (0.73 to 1.12)
P value	0.731	0.245	0.744	0.374
CYP3A4 (rs4646440)				
GG	409.56 (357.12 to 462.01)	303.30 (254.50 to 352.11)	68.60 (57.13 to 80.07)	0.90 (0.76 to 1.03)
GA	437.91 (380.06 to 495.77)	328.43 (274.60 to 382.27)	69.38 (56.73 to 82.04)	0.74 (0.59 to 0.88)
AA	372.74 (230.18 to 515.30)	245.70 (113.04 to 378.36)	68.10 (36.91 to 99.28)	0.69 (0.33 to 1.06)
P value	0.621	0.485	0.995	0.228
CYP3A5 (rs4646450)				
GG	378.49 (322.52 to 434.47)	273.48 (221.07 to 325.88)	64.82 (52.57 to 77.08)	0.89 (0.75 to 1.03)
GA	456.07 (402.26 to 509.88)	340.89 (290.51 to 391.27)	69.98 (58.20 to 81.76)	0.75 (0.61 to 0.89)
AA	401.30 (279.67 to 522.94)	296.38 (182.51 to 410.26)	79.98 (53.35 to 106.61)	0.74 (0.43 to 1.05)
P value	0.144	0.190	0.573	0.344
CYP3A5 (rs4646453)				
CC	412.99 (358.28 to 467.70)	289.44 (238.54 to 340.35)	67.36 (55.43 to 79.29)	0.84 (0.70 to 0.98)
CA	430.75 (376.57 to 484.93)	336.34 (285.92 to 386.75)	69.48 (57.66 to 81.29)	0.80 (0.66 to 0.93)
AA	345.44 (196.66 to 494.22)	220.86 (82.42 to 359.30)	71.63 (39.19 to 104.07)	0.69 (0.31 to1.08)
P value	0.565	0.205	0.952	0.744
CYP2D6*10 (rs1065852)				
AA	453.42 (359.54 to 547.30)	235.60 (148.33 to 322.87)	84.20 (63.91 to 104.50)	0.54 (0.30 to 0.77)
AG	416.66 (364.52 to 468.80)	314.44 (265.97 to 362.91)	62.31 (51.04 to 73.58)	0.83 (0.70 to 0.96)
GG	399.52 (334.22 to 464.82)	331.66 (270.95 to 392.36)	71.00 (56.88 to 85.11)	0.92 (0.75 to 1.08)
P value	0.647	0.191	0.176	**0.032**
CYP2D6*10 (rs1135840)				
GG	430.45 (373.85 to 487.06)	316.85 (263.96 to 369.7)	73.51 (61.21 to 85.81)	0.72 (0.58 to 0.87)
GC	413.42 (356.20 to 470.64)	297.87 (244.41 to 351.34)	64.78 (52.34 to 77.21)	0.89 (0.74 to 1.03)
CC	385.25 (280.22 to 490.27)	299.92 (201.79 to 398.05)	66.56 (43.73 to 89.38)	0.83 (0.57 to 1.10)
P value	0.749	0.876	0.607	0.274
**400 mg/day**				
CYP2C8 (rs7910936)				
TT	489.54 (355.09 to 624.00)	278.56 (172.45 to 384.67)	60.62 (40.53 to 80.72)	0.82 (0.64 to 1.01)
TC	745.21 (659.35 to 831.06)	452.43 (384.67 to 520.19)	97.71 (84.88 to 110.55)	0.60 (0.48 to 0.71)
CC	753.97 (625.96 to 881.99)	519.32 (418.28 to 620.35)	100.90 (81.77 to 120.04)	0.73 (0.55 to 0.90)
P value	**0.004**	**0.004**	**0.005**	0.100
CYP2C8 (rs10882521)				
GG	689.87 (574.72 to 805.02)	452.53 (361.21 to 543.85)	93.42 (76.27 to 110.58)	0.74 (0.58 to 0.89)
GT	765.34 (679.97 to 850.71)	472.41 (404.71 to 540.12)	100.66 (87.94 to 113.38)	0.61 (0.49 to 0.73)
TT	445.24 (286.98 to 603.50)	249.64 (124.13 to 375.15)	50.11 (26.53 to 73.68)	0.80 (0.58 to 1.01)
P value	**0.003**	**0.009**	**0.001**	0.224
CYP2C8 (rs17110453)				
AA	742.37 (644.35 to 840.39)	439.59 (361.89 to 517.29)	90.93 (76.22 to 105.64)	0.69 (0.56 to 0.83)
AC	698.18 (602.17 to 794.19)	456.00 (379.89 to 532.11)	95.41 (81.00 to109.81)	0.67 (0.54 to 0.80)
CC	506.48 (326.46 to 686.49)	324.64 (181.94 to 467.33)	74.09 (47.07 to101.10)	0.69 (0.45 to 0.94)
P value	0.079	0.272	0.391	0.958
CYP3A4*1G (rs2242480)				
CC	702.69 (619.77 to 785.61)	441.30 (375.74 to 506.85)	86.94 (74.55 to 99.33)	0.60 (0.49 to 0.71)
CT	675.93 (565.30 to 786.57)	422.03 (334.57 to 509.49)	93.58 (77.05 to 110.11)	0.83 (0.68 to 0.98)
TT	721.47 (457.60 to 985.33)	411.30 (202.71 to 619.90)	111.22 (71.80 to 150.65)	0.63 (0.28 to 0.98)
P value	0.911	0.921	0.466	0.056
CYP3A4 (rs2246709)				
GG	692.61 (549.96 to 835.25)	403.19 (290.52 to 515.86)	80.48 (59.13 to 101.83)	0.72 (0.53 to 0.91)
GA	680.68 (583.15 to 778.22)	440.81 (363.77 to 517.85)	93.51 (78.91 to108.11)	0.72 (0.58 to 0.85)
AA	701.91 (597.06 to 806.76)	437.09 (354.26 to 519.91)	93.44 (77.75 to109.13)	0.62 (0.48 to 0.76)
P value	0.958	0.854	0.566	0.546
CYP3A4 (rs4646440)				
GG	688.44 (609.11 to 767.77)	434.83 (372.04 to 497.62)	84.39 (72.57 to 96.21)	0.62 (0.51 to 0.73)
GA	713.44 (589.83 to 837.05)	430.17 (332.34 to 528.01)	100.43 (82.01 to118.85)	0.82 (0.65 to 0.98)
AA	721.68 (448.42 to 994.94)	404.03 (187.74 to 620.31)	110.20 (69.48 to 150.92)	0.62 (0.26 to 0.99)
P value	0.930	0.963	0.216	0.141
CYP3A5 (rs4646450)				
GG	711.04 (626.08 to 796.00)	454.29 (387.15 to 521.43)	88.72 (76.03 to 101.41)	0.61 (0.50 to 0.72)
GA	638.90 (534.94 to 742.86)	389.84 (307.69 to 471.99)	88.92 (73.39 to 104.45)	0.80 (0.66 to 0.93)
AA	861.16 (580.25 to 1142.07)	484.90 (262.91 to 706.88)	121.79 (79.83 to 163.75)	0.63 (0.25 to 1.01)
P value	0.271	0.435	0.323	0.130
CYP3A5 (rs4646453)				
CC	700.83 (618.89 to 782.77)	439.55 (374.81 to 504.28)	84.99 (72.78 to 97.20)	0.62 (0.51 to 0.73)
CA	686.21 (576.80 to 795.62)	414.89 (328.45 to 501.32)	97.95 (81.64 to114.25)	0.79 (0.65 to 0.94)
AA	560.31 (227.82 to 892.80)	417.51 (154.83 to 680.19)	105.20 (55.64 to154.75)	0.67 (0.23 to 1.12)
P value	0.719	0.901	0.385	0.166
CYP2D6*10 (rs1065852)				
AA	776.75 (627.17 to 926.33)	356.89 (239.26 to 474.52)	107.65 (85.33 to 129.98)	0.44 (0.24 to 0.64)
AG	646.89 (561.16 to 732.63)	414.16 (346.74 to 481.58)	84.69 (71.90 to 97.50)	0.72 (0.61 to 0.84)
GG	722.18 (598.83 to 845.52)	513.08 (416.08 to 610.07)	89.91 (71.50 to 108.32)	0.76 (0.60 to 0.92)
P value	0.282	0.105	0.216	**0.036**
CYP2D6*10 (rs1135840)				
GG	676.08 (582.02 to 770.13)	408.29 (333.90 to 482.67)	85.74 (71.64 to 99.85)	0.68 (0.55 to 0.80)
GC	712.98 (607.22 to 818.74)	424.96 (341.31 to 508.61)	94.75 (78.89 to 110.60)	0.69 (0.54 to 0.83)
CC	643.34 (465.50 to 821.17)	537.60 (396.94 to 678.26)	90.93 (64.30 to 117.60)	0.69 (0.45 to 0.93)
P value	0.778	0.271	0.714	0.990

Adjusted by weight, age and duration of HCQ treatment.

Significant p values are in bold.

DCQ, desethylchloroquine; DHCQ, desethylhydroxychloroquine; HCQ, hydroxychloroquine; SNP, single nucleotide polymorphism.

### Relationship between gene polymorphisms and the optimal blood HCQ concentration

To explore the relationship between SNPs and the optimal blood concentration (HCQ=559.67 ng/mL), we took logistic regression analysis in different dose groups (table 4). We found the CC genotype of CYP2C8 (rs7910936) was more likely to achieve the optimal blood concentration in HCQ 200 mg/day group (OR=9.48; 95% CI=1.25 to 71.85; p=0.030), indicating patients with the CYP2C8 (rs7910936) CC genotype would be able to achieve the effective threshold concentration when taking HCQ 200 mg/day. Besides, CYP2D6*10 (rs1065852) GG genotype was more likely to reach the optimal blood concentration in HCQ 400 mg/day group (OR=3.10; 95% CI=1.00 to 9.59; p=0.049), suggesting patients with the CYP2D6*10 (rs1065852) GG genotype could achieve the optimal blood concentration when taking HCQ 400 mg/day.

**Table 4:** The associations between SNPs and the optimal blood HCQ concentration

	200 mg/day group		400 mg/day group	
	**OR (95% CI**)	**P value**	**OR (95% CI**)	**P value**
CYP2C8 (rs7910936)				
TT	Reference		Reference	
TC	6.83 (0.89 to 52.24)	0.064	1.13 (0.42 to 3.01)	0.809
CC	9.48 (1.25 to 71.85)	**0.030**	2.03 (0.52 to 7.97)	0.310
CYP2C8 (rs10882521)				
GG	Reference		Reference	
GT	0.80 (0.23 to 2.82)	0.724	1.41 (0.45 to 4.46)	0.561
TT	0.82 (0.09 to 7.79)	0.864	0.30 (0.06 to 1.39)	0.123
CYP2D6*10 (rs1065852)				
AA	Reference		Reference	
AG	0.47 (0.16 to 1.39)	0.172	0.67 (0.31 to 1.46)	0.314
GG	0.90 (0.29 to 2.86)	0.863	3.10 (1.00 to 9.59)	**0.049**

OR adjusted by weight, age and duration of HCQ treatment.

Significant p values are in bold.

HCQ, hydroxychloroquine; SNPs, single nucleotide polymorphisms.

### Relationship between gene polymorphisms and SLEDAI

SLEDAI decreased to 4.16±2.34 in 200 mg/day group and 3.67±2.04 in 400 mg/day group after taking HCQ for more than 3 months. Patients had lower disease activity with HCQ 400 mg/day (p=0.014). We investigated the relationship between different genotypes and the decrease of SLEDAI after HCQ treatment (shown as ΔSLEDAI), and found that ΔSLEDAI was lower in patients with the CYP2D6*10 (rs1065852) AG genotype in 400 mg/day group (p=0.013) (online supplemental table 6). For CYP2C8 (rs7910936 and rs10882521), we did not find an association with ΔSLEDAI.

## Discussion

Stable and effective blood concentration of HCQ is essential for preventing SLE flares, reducing complications risk and improving relapse rates.^12^ In this study, SLEDAI was relatively accurate in evaluating HCQ efficacy with the AUC up to 0.75. Significant improvement in the patients’ condition was observed upon reaching the optimal blood drug concentration. Combined with the results of the stratified analysis, the optimal blood concentration of HCQ measured approximately 12–18 hours after the last dosage may be between 500 and 600 ng/mL.

Some previous studies in Europe, the USA and some other countries have proposed effective blood concentration thresholds for HCQ. For example, Costedoat-Chalumeau *et al* suggested 1000 ng/mL as the target concentration of HCQ in the whole blood of French patients with SLE,^22^ Garg *et al* supported the clinical significance and statistical significance of HCQ levels ≥750 ng/mL according to their study, mainly European populations in a meta-analysis,^9^ and Cunha *et al* suggested that an HCQ target level to prevent flares should be at least 600 ng/mL in the UK.^23^ Compared with these studies, our study’s effective concentration threshold of HCQ was significantly lower. For one thing, we knew that HCQ concentration was associated with the dose administered, and body weight significantly affected the clearance of HCQ.^24^ In addition, pharmacogenetic polymorphisms can affect the rate of HCQ metabolism in vivo and may be an important reason for the differences in blood levels.

In this study, we explored the association between CYP450 polymorphisms and blood concentrations of HCQ and its metabolites in Chinese patients with SLE. CYP2C8 is a major human hepatic P450, accounting for approximately 7% of the total microsomal CYP content in the liver.^25^ The CYP2C8 polymorphism is thought to play a role in chloroquine metabolism, but the effect on HCQ is unclear.^15^ This study found polymorphisms of CYP2C8 (rs7910936 and rs10882521) associated with HCQ and its metabolites. The C allele of CYP2C8 (rs7910936) was significantly related to the increase in blood drug concentrations, and the CYP2C8 (rs10882521) TT genotype was associated with lower blood drug concentrations. To date, the impact of CYP2C8 (rs7910936 and rs10882521) polymorphisms on drug metabolism in European, American and other populations remains unknown, and existing research in the Chinese population requires further validation.^26^ Our findings indicated that the CYP2C8 polymorphism might be an essential factor affecting HCQ and its metabolites concentrations and deserved further study.

The CYP2D6 gene is highly polymorphic, with more than 70 alleles.^24^ The major CYP2D6 alleles in Europeans are CYP2D6*3, CYP2D6*4 and CYP2D6*5, which are very rare in Asians.^27^ CYP2D6*10 is the most common CYP2D6 allele in Asian populations, with a 35–55% frequency in Chinese, Japanese and Korean populations. The CYP2D6*10 allele significantly reduces enzyme activity due to reduced protein stability, disrupted substrate recognition and reduced substrate–enzyme affinity, leading to an increase in blood drug concentration.^28–30^ Our results were relatively consistent with previous studies. Although there was no significant difference in HCQ concentration, variants in alleles of CYP2D6*10 (rs1065852) made patients more likely to reach the optimal blood concentration in the 400 mg/day group. In addition, Lee *et al* suggested that the DHCQ:HCQ ratio may be a good predictor of a patient’s response to HCQ treatment,^14^ and our results confirmed the polymorphism of CYP2D6*10 (rs1065852) can influence the equilibrium state between HCQ and DHCQ concentrations.

CYP3A family CYP3A4 and CYP3A5 are abundantly expressed in adults^31–33^ and highly polymorphic.^34^ Studies suggested that polymorphism of CYP4/5 may affect the catalytic activity.^35–37^ CYP3A4 (rs2242480 and rs4646440), common in the Chinese population, have more than 20% MAF and can increase the catalytic activity of CYP3A4.^38 39^ However, in our study, CYP3A4 and CYP3A5 were not found to correlate with blood concentrations. Future studies with larger sample sizes may be useful to investigate any associations between the CYP3A4/5 SNPs and HCQ metabolism.

We also further associated CYP450 polymorphisms with treatment outcomes and explored the effect of polymorphisms on the optimal blood HCQ concentration and SLEDAI, and found that the CC genotype of CYP2C8 (rs7910936) was more likely to achieve the optimal blood concentration with HCQ 200 mg/day and the CYP2D6*10 (rs1065852) GG genotype was more likely to reach the optimal blood concentration with HCQ 400 mg/day. In addition, SLEDAI decreased more in patients with the CYP2D6*10 (rs1065852) GG and AA genotypes in 400 mg/day group after treatment. Therefore, we hypothesised that patients with SLE with the CYP2D6*10 (rs1065852) GG genotype might have good treatment responses when taking HCQ 400 mg/day, although the CIs were wide which implied a high degree of variability and uncertainty in the data. We think this study makes a valuable contribution to the field because these findings may provide a plausible explanation for interindividual variations of HCQ concentrations.

In addition, we found that after a period of HCQ use, the patients’ condition improved significantly. The patients’ laboratory indices improved (eg, the patient’s haematological indices of WBC and PLT; immunological indices of C3 and C4, etc) and SLEDAI decreased. Patients taking 400 mg/day HCQ had lower SLEDAI, which may be explained by higher HCQ blood levels. Consistent with previous studies, HCQ blood levels were closely related to the treatment response.

In this study, we selected multiple SNPs to comprehensively investigate the association between the polymorphisms of CYP450 enzymes and the blood concentrations of HCQ and its metabolites. We determined the optimal blood concentration of HCQ and further explored the associations between SNPs and the treatment effects. However, our study had certain limitations. First, although the study considered whether glucocorticoids or immunosuppressants were being used, it did not analyse specific drug usage (quantity, dosage, duration of use, combination use, etc), which may affect the accuracy of the results. Second, this study was limited by patient compliance. Although we have excluded some low concentration patients, some patients may still have forgotten or missed medication. Third, the dosing recommendations did not take into account weight, nor the possibility of using an intermediate dosage such as 300 mg/day. Fourth, bias may be introduced with the timing of SLEDAI assessments. Fifth, as a single-centre study with a limited representation of Chinese patients with SLE, research of multicentre, extensive sample studies and validation were needed.

## Conclusions

The blood concentration of HCQ is negatively correlated with SLEDAI. Our results suggest that the optimal blood concentration of HCQ measured approximately 12–18 hours after the last dosage may be between 500 and 600 ng/mL in Chinese patients with SLE. We found the TC and CC genotypes of CYP2C8 (rs7910936) were significantly related to the increase in blood HCQ concentrations, and the CYP2C8 (rs10882521) TT genotype was associated with lower blood HCQ concentrations. Besides, polymorphisms of CYP2D6*10 (rs1065852) can influence the equilibrium state between HCQ and DHCQ concentrations. Genotypical testing of patients and regular monitoring of blood levels are recommended for optimising HCQ dosage management in Chinese patients with SLE.

## Data Availability

Data are available upon reasonable request.
