# Supplementary material for: Genotype-guided new approach for dose optimisation of hydroxychloroquine administration in Chinese patients with SLE
Source: Lupus Sci Med. 2023 Nov 22;10(2):e000997. doi: 10.1136/lupus-2023-000997 (PMC10668244; doi:10.1136/lupus-2023-000997)
Supplement: Supplementary data [file lupus-2023-000997supp001.pdf]

Supplementary Table S1 Comparison of clinical characteristics before and after administration of HCQ for more than 3 months

Variable	Before treatment	After treatment	P
WBC, counts $\times10^9$/L	5.23 ± 2.85	6.12 ± 3.05	**< 0.001**
PLT, counts $\times10^9$/L	167.48 ± 95.24	180.28 ± 92.00	**< 0.001**
RBC, counts $\times10^{12}$/L	3.60 ± 0.80	3.58 ± 0.76	0.477
CRP, mg/L	11.72 ± 19.35	8.13 ± 15.54	**< 0.001**
C3, g/L	0.87 ± 0.35	0.94 ± 0.30	**< 0.001**
C4, g/L	0.16 ± 0.09	0.18 ± 0.09	**< 0.001**
IgG, g/L	14.68 ± 6.33	12.41 ± 5.56	**< 0.001**
IgA, g/L	2.72 ± 1.59	2.55 ± 1.45	**< 0.001**
IgM, g/L	1.43 ± 1.98	1.36 ± 1.93	0.087
ESR, mm/h	40.54 ± 29.67	27.66 ± 21.89	**< 0.001**
SLEDAI	8.01 ± 3.74	3.88 ± 2.19	**< 0.001**
ANA positive, no. (%)	433 (88.55)	430 (87.93)	0.766
Anti-ds DNA positive, no. (%)	216 (44.17)	148 (30.27)	**< 0.001**
Receiving concomitant glucocorticoids, no. (%)	371 (75.87)	344 (70.35)	0.052
Receiving concomitant immunosuppressants, no. (%)	221 (45.19)	198 (40.49)	0.137

Values are mean ± SD or number of patients with percentage in parentheses.
Significant p values are in bold.

Xie H, *et al. Lupus Sci Med* 2023; 10:e000997. doi: 10.1136/lupus-2023-000997

Supplementary Table S2 Comparison of patients' characteristics in effective and ineffective groups after HCQ treatment

	Effective group (n = 345)	Ineffective group (n = 144)	P
Age, years	43.09 ± 15.78	46.77 ± 14.16	**0.012**
Weight, kg	59.31 ± 13.07	59.98 ± 10.61	0.581
BMI, kg/m^2	22.47 ± 4.11	22.83 ± 3.62	0.360
Duration of HCQ treatment, months	12.88 ± 10.90	10.51 ± 10.36	**0.027**
HCQ dose	321.16 ± 97.88	287.50 ± 99.56	**0.001**
200 mg/d, no. (%)	136 (39.42)	81 (56.25)	
400 mg/d, no. (%)	209 (60.58)	63 (43.75)	
SLEDAI	2.88 ± 1.38	6.28 ± 1.88	**< 0.001**
Ever used glucocorticoids, no. (%)	304 (88.12)	122 (84.72)	0.307
Ever used immunosuppressive drugs, no. (%)	192 (55.65)	64 (44.44)	**0.024**
[HCQ], ng/mL	667.05 ± 496.24	335.38 ± 261.15	**< 0.001**
[DHCQ], ng/mL	433.07 ± 389.29	241.18 ± 277.70	**< 0.001**
[DCQ], ng/mL	90.76 ± 78.47	57.60 ± 52.72	**< 0.001**
[DHCQ]: [HCQ]	0.69 ± 0.53	0.86 ± 1.02	**0.049**
Anti-ds DNA positive, no. (%)	93 (26.96)	55 (38.19)	**0.014**
ANA positive, no. (%)	304 (88.12)	126 (87.50)	0.849
WBC, $\times 10^9/L$	6.12 ± 2.97	6.12 ± 3.24	0.985
PLT, $\times 10^9/L$	188.68 ± 88.96	160.14 ± 96.27	**0.002**
RBC, $\times 10^{12}/L$	3.59 ± 0.76	3.56 ± 0.77	0.732
Neutrophil percent, %	69.23 ± 12.74	67.38 ± 13.19	0.149
Lymphocyte percent, %	22.00 ± 10.95	24.47 ± 11.32	**0.025**
Alanine transaminase, U/L	24.45 ± 33.66	25.02 ± 25.38	0.855
Aspartate aminotransferase, U/L	23.81 ± 28.46	23.12 ± 17.05	0.789
Albumin, g/L	35.09 ± 5.14	35.01 ± 5.45	0.873
Glucose, mmol/L	4.64 ± 2.94	4.64 ±1.09	0.994
Total cholesterol, mmol/L	4.50 ± 1.44	4.47 ± 1.28	0.818
Triglyceride, mmol/L	1.69 ± 1.07	1.84 ± 1.24	0.178
High-density lipoprotein cholesterol, mmol/L	1.31 ± 0.50	1.25 ± 0.44	0.198
Low-density lipoprotein cholesterol, mmol/L	2.46 ± 1.08	2.43 ± 0.94	0.769
Blood urea nitrogen, mmol/L	7.93 ± 5.98	7.85 ± 6.43	0.896
Creatinine, umol/L	90.58 ± 114.51	83.95 ± 106.32	0.552
eGFR, $mL/min/1.73m^2$	114.35 ± 52.55	114.16 ± 51.02	0.971
CRP, mg/L	7.31 ± 10.23	10.11 ± 23.81	0.177
C3, g/L	0.93 ± 0.28	0.95 ± 0.34	0.633
C4, g/L	0.18 ± 0.08	0.19 ± 0.11	0.347
IgG, g/L	12.16 ± 5.64	13.00 ± 5.35	0.128
IgA, g/L	2.48 ± 1.44	2.71 ± 1.47	0.105
IgM, g/L	1.21 ± 1.21	1.74 ± 2.99	**0.039**
ESR, mm/h	25.82 ± 20.25	32.09 ± 24.92	**0.008**

eGFR, estimated glomerular filtration rate.

The effective group of HCQ was defined by SLEDAI ≤ 4, and ineffective group was SLEDAI > 4.

Significant p values are in bold.

Xie H, *et al. Lupus Sci Med* 2023; 10:e000997. doi: 10.1136/lupus-2023-000997

Supplementary Table S3 ROC analysis of [HCQ], [DHCQ], [DCQ], and [DHCQ]: [HCQ] in predicting efficacy

	AUC	P	Specificity	Sensitivity	Cutoff value
[HCQ]	0.75	**< 0.001**	0.89	0.51	559.67 ng/mL
[DHCQ]	0.68	**< 0.001**	0.63	0.69	183.12 ng/mL
[DCQ]	0.63	**< 0.001**	0.74	0.50	64.72 ng/mL
[DHCQ]: [HCQ]	0.53	0.359	0.55	0.59	0.50

The effective group of HCQ was defined by SLEDAI ≤ 4, and ineffective group was SLEDAI > 4.
Significant p values are in bold.

Supplementary Table S4 Comparison of clinical indicators between high and low concentration groups after HCQ treatment

Variable	High concentration group (n = 193)	Low concentration group (n = 296)	P
WBC, counts $\times 10^9$/L	6.41 ± 3.14	5.93 ± 2.98	0.088
PLT, counts $\times 10^9$/L	203.44 ± 93.94	165.18 ± 87.62	**< 0.001**
RBC, counts $\times 10^{12}$/L	3.60 ± 0.76	3.56 ± 0.77	0.590
CRP, mg/L	7.55 ± 9.75	8.52 ± 18.36	0.499
C3, g/L	0.97 ± 0.28	0.92 ± 0.31	0.059
C4, g/L	0.20 ± 0.08	0.17 ± 0.09	**0.001**
IgG, g/L	11.90 ± 5.94	12.74 ± 5.29	0.102
IgA, g/L	2.57 ± 1.70	2.53 ± 1.27	0.798
IgM, g/L	1.07 ± 1.01	1.56 ± 2.32	**0.001**
ESR, mm/h	27.09 ± 21.41	28.04 ± 22.23	0.639
SLEDAI, no. (%)			**< 0.001**
SLEDAI ≤ 4	177 (91.71)	168 (56.76)	
SLEDAI > 4	16 (8.29)	128 (43.24)	
ANA positive, no. (%)	165 (85.49)	265 (89.53)	0.181
Anti-ds DNA positive, no. (%)	44 (22.80)	104 (35.14)	**0.004**

Values are mean ± SD or number of patients with percentage in parentheses.
Significant p values are in bold.

Xie H, *et al. Lupus Sci Med* 2023; 10:e000997. doi: 10.1136/lupus-2023-000997

Supplementary Table S5 The results of the optimal blood HCQ concentration, stratified by concomitant medications

Concomitant medications	AUC	P	Specificity	Sensitivity	Cutoff value (ng/mL)
With immunosuppressants (n = 256)	0.79	**< 0.001**	0.90	0.58	502.10
Mycophenolate mofetil (n = 66)	0.70	**0.009**	0.81	0.71	523.89
Tacrolimus (n = 72)	0.77	**< 0.001**	0.96	0.55	535.10
With glucocorticoids (n = 426)	0.67	**< 0.001**	0.82	0.55	540.71
Without concomitant medications (n = 49)	0.80	**< 0.001**	0.95	0.67	561.12

The effective group of HCQ was defined by SLEDAI ≤ 4, and ineffective group was SLEDAI > 4.
Significant p values are in bold.

BMJ Publishing Group Limited (BMJ) disclaims all liability and responsibility arising from any reliance placed on this supplemental material which has been supplied by the author(s)

Supplementary Table S6 The associations between SNPs and SLEDAI after HCQ treatment

	200 mg/d group	400 mg/d group
	**ΔSLEDAI (95% CI)**	**ΔSLEDAI (95% CI)**
CYP2C8 (rs7910936)		
TT	3.04 (1.94 -4.14)	4.23 (3.37-5.09)
TC	4.43 (3.72-5.13)	4.21 (3.66-4.76)
CC	3.93 (2.98-4.88)	4.41 (3.59-5.23)
P	0.120	0.922
CYP2C8 (rs10882521)		
GG	3.43 (2.57-4.29)	4.43 (3.69-5.16)
GT	4.62 (3.89-5.34)	4.39 (3.85-4.94)
TT	3.35 (2.22-4.48)	3.53 (2.52-4.55)
P	0.059	0.305
CYP2D6*10 (rs1065852)		
AA	4.33 (3.06-5.59)	5.45 (4.52-6.38)
AG	4.05 (3.35-4.76)	3.84 (3.30-4.40)
GG	3.67 (2.79-4.55)	4.34 (3.57-5.11)
P	0.658	**0.013**

Adjusted by weight, age, and duration of HCQ treatment.

Significant p values are in bold.
