# Supplementary figures and images for: Genotype-guided new approach for dose optimisation of hydroxychloroquine administration in Chinese patients with SLE
Source: Lupus Sci Med. 2023 Nov 22;10(2):e000997. doi: 10.1136/lupus-2023-000997 (PMC10668244; doi:10.1136/lupus-2023-000997)

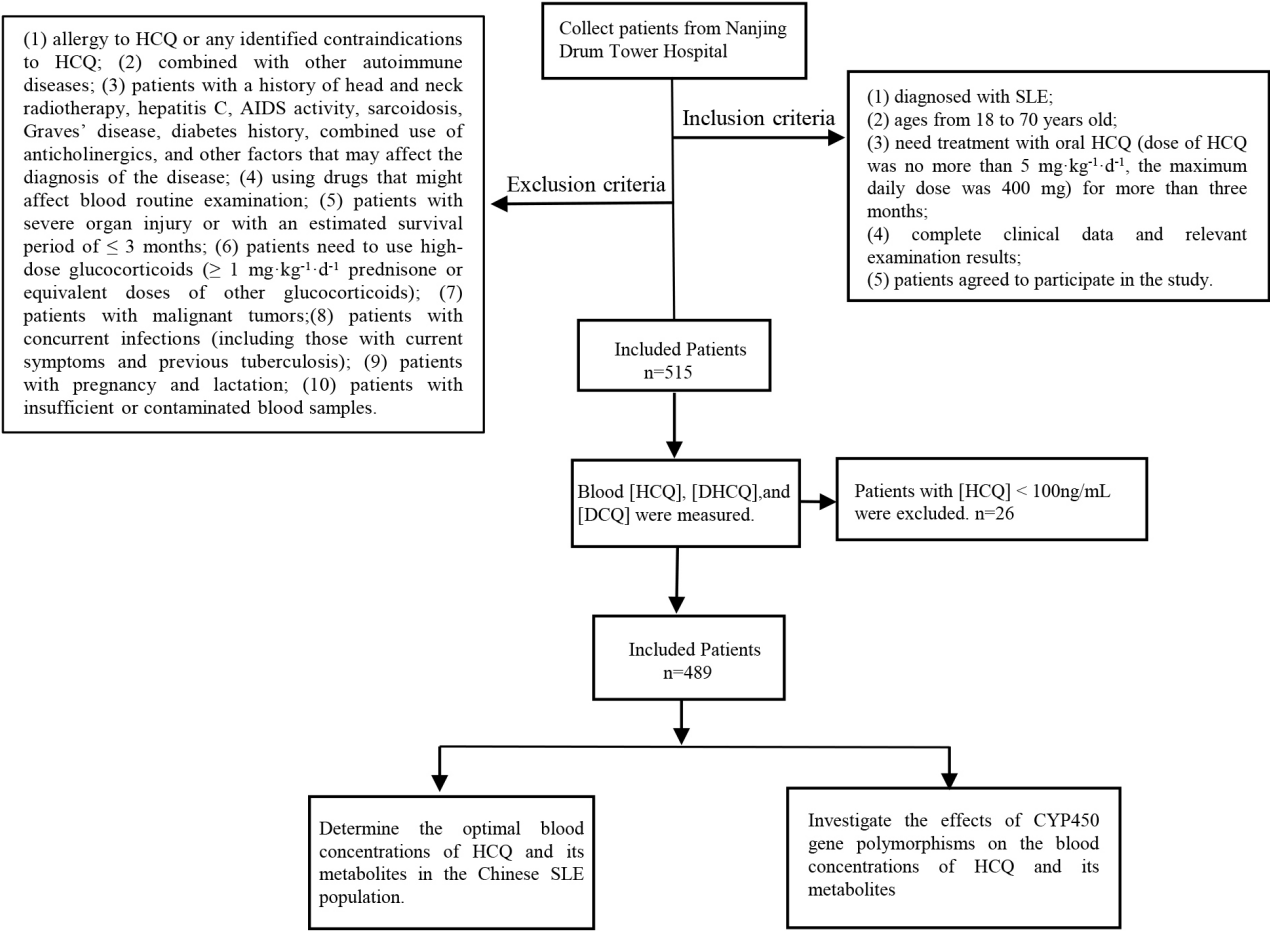

Supplement: Supplementary data [file lupus-2023-000997supp002.pdf]

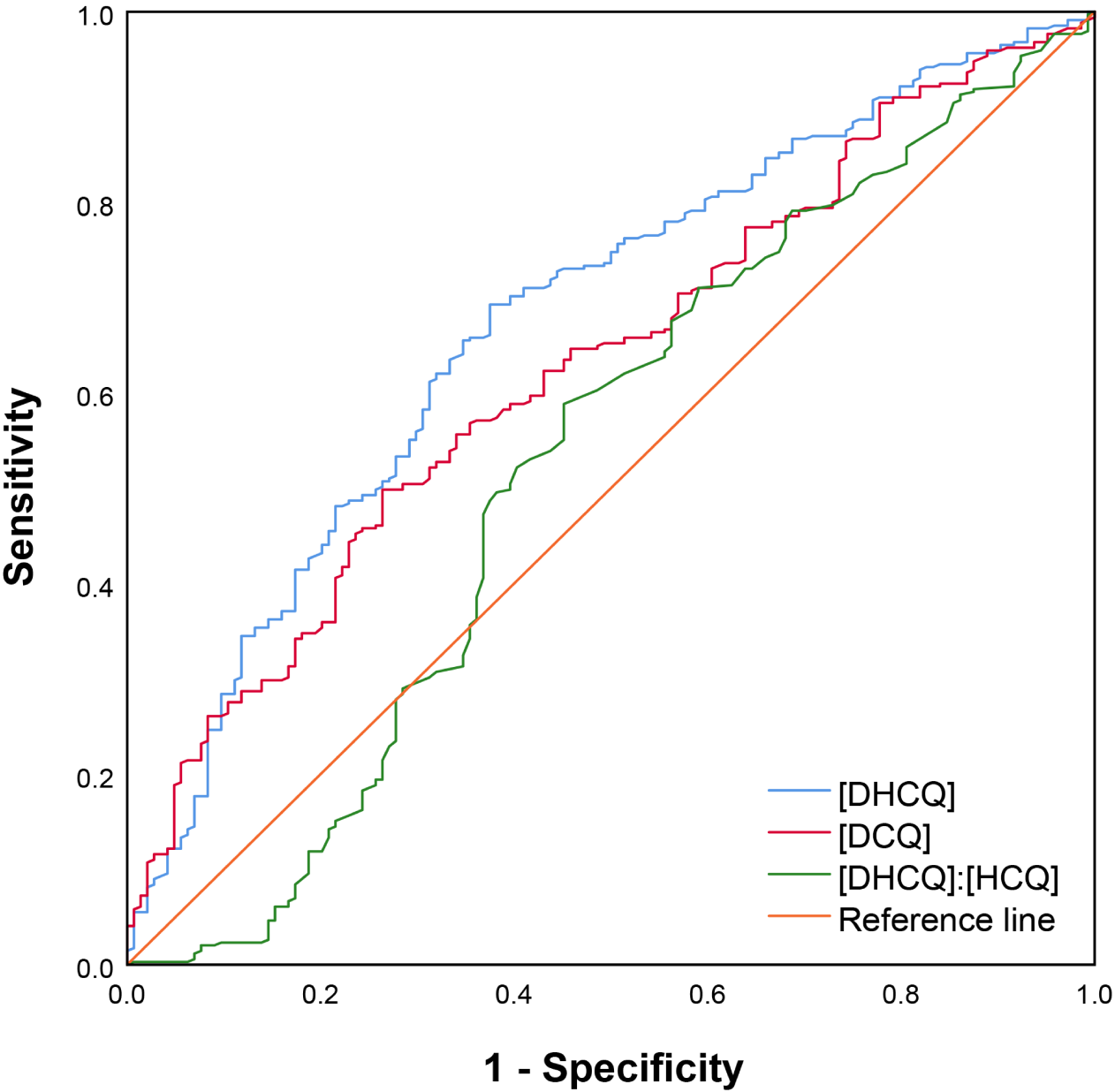

Supplement: Supplementary data [file lupus-2023-000997supp003.pdf]

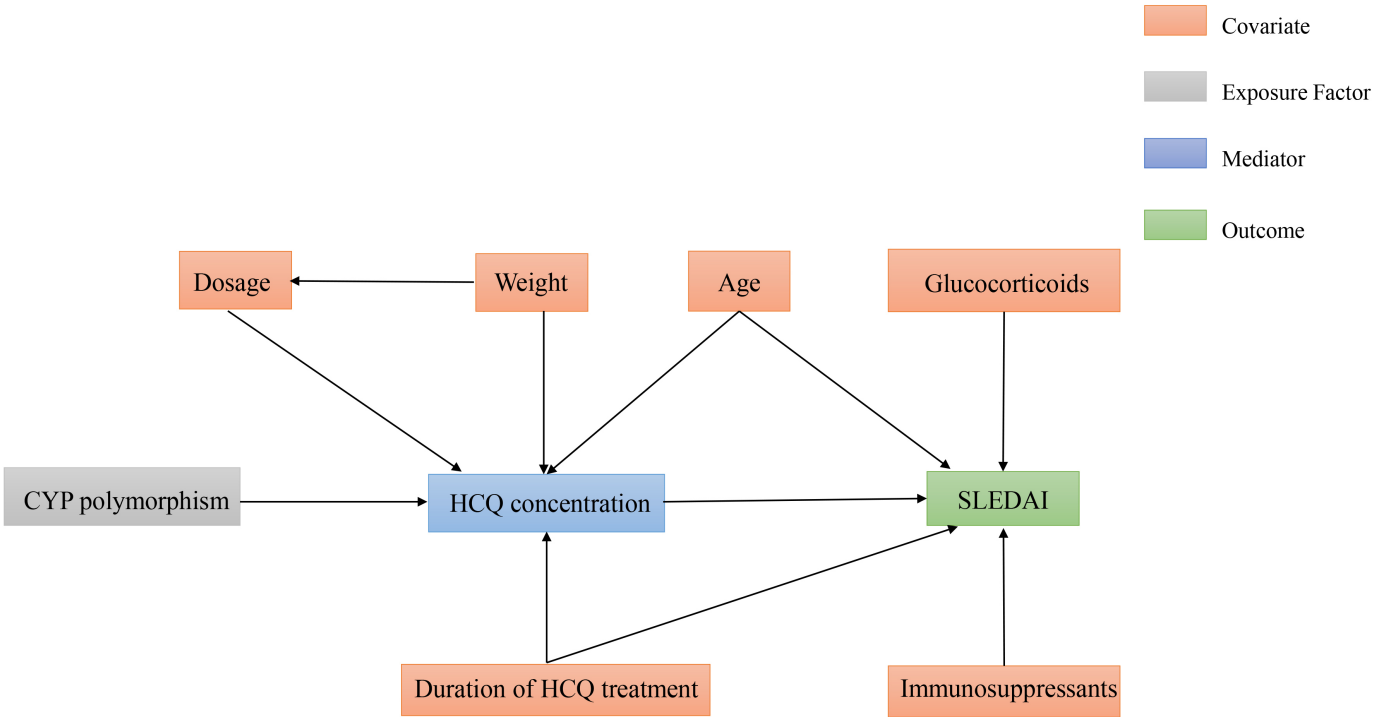

Supplement: Supplementary data [file lupus-2023-000997supp004.pdf]
